# Axillary and elbow lymph node metastasis arising after complete excision of microcystic adnexal carcinoma of a hand: A rare presentation

**DOI:** 10.1016/j.ijscr.2019.10.023

**Published:** 2019-10-17

**Authors:** Kobkool Chakrapan Na Ayudhya, Vichack Chakrapan Na Ayudhya, Panat Tipsuwannakul, Sarun Thongvitokomarn, Vorapatu Tangsirapat, Panutchaya Kongon, Juthamas Thananon, Sirirat Sookpotarom, Paiboon Sookpotarom, Paisarn Vejchapipat

**Affiliations:** a Department of Surgery, Panyananthaphikkhu Chonprathan Medical Center, Srinakharinwirot University, Nonthaburi, 11120, Thailand; b Department of Radiology, Panyananthaphikkhu Chonprathan Medical Center, Srinakharinwirot University, Nonthaburi, 11120, Thailand; c Department of Dentistry, Panyananthaphikkhu Chonprathan Medical Center, Srinakharinwirot University, Nonthaburi, 11120, Thailand; d Department of Surgery, Faculty of Medicine, Chulalongkorn University, Bangkok, 10330, Thailand

**Keywords:** Microcystic adnexal carcinoma, Distant metastasis, Recurrence

## Abstract

**Introduction:**

Microcystic adnexal carcinoma (MAC), a malignant transformation of adnexal structures, constitutes a rare locally aggressive malignancy of skin. Generally, the disease is associated with local invasion and distant metastases are extremely rare. We presented a case of MAC with distant metastasis after adequate wide excision.

**Presentation of case:**

A 65-year-old man presented with a scar-like lesion at his left hand’s middle finger. As pathologic result from incisional biopsy confirmed MAC, the patient was then treated with ray amputation. Eight years later, there was a presence of scar changes. There was a palpable node at medial epicondyle and presence of enlarged lymph nodes at axilla by imaging with Computed tomography (CT) scan. Following left hand amputation and node dissection at elbow and axillary region, pathologic examination confirmed recurrence and metastasis of MAC. He went through adjuvant radiation with a complete response. At present, two years after surgery, he remains in complete remission.

**Discussion:**

The presence of a recurrent MAC with distant lymph node metastasis after primary surgery is rare. The metastatic route as presented with the positive nodes at elbow and axilla could be explained the spreading via lymphatic system.

**Conclusion:**

MAC arising at a hand may require wider excision in order to achieve best result. Although this malignancy is a slowly progressive disease, distant metastasis should always be concerned, especially when there are suspicious presentations. CT scan will render more details and make more precise diagnosis in the suspicious situation.

## Highlights

• MAC arising at a hand may require wider excision in order to achieve best result.
• The metastatic route could be explained the spreading via lymphatic system.
• CT scan renders more details and more precise diagnosis in a suspicious situation.

## 1 Introduction

Malignant cutaneous adnexal neoplasms (MCANs) are rare malignancy of skin that derived from adnexal structures including sweat glands, sebaceous glands, and hair follicles. Microcystic adnexal carcinoma (MAC), a subtype of MCANs and generally believed to derive from eccrine glands in origin, is extremely rare [1]. As the natural history of the disease is a very slowly progressive disease, distant metastasis particularly to lymph nodes seldom occurs [2]. Generally, wide excision achieving free margins yields an excellent result [[3], [4], [5], [6]]. Therefore, the report of our patient who presented with distant lymph node metastasis following an adequate surgical removal is an unusual event. To our knowledge, the disease course of MAC like this has never been reported before in the literature.

This work is compliant with the SCARE checklist, and also, has been reported in line with the SCARE criteria [7].

## 2 Presentation of case

**Fig. 1:**
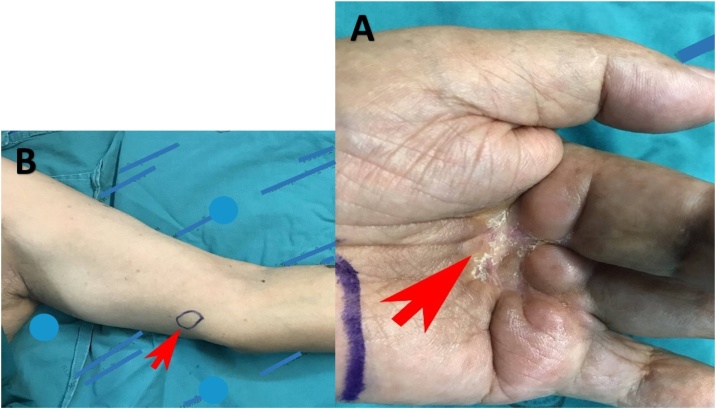
A, A scar changes at amputated stump; the scar became harder and more contracted into the proximal part (large red arrow); B, The lymph node enlargement at left medial epicondyle (small red arrow). *Remark: All markers revealing hospital’s name in fig. 1 are have already been covered with bars.

**Fig. 2:**
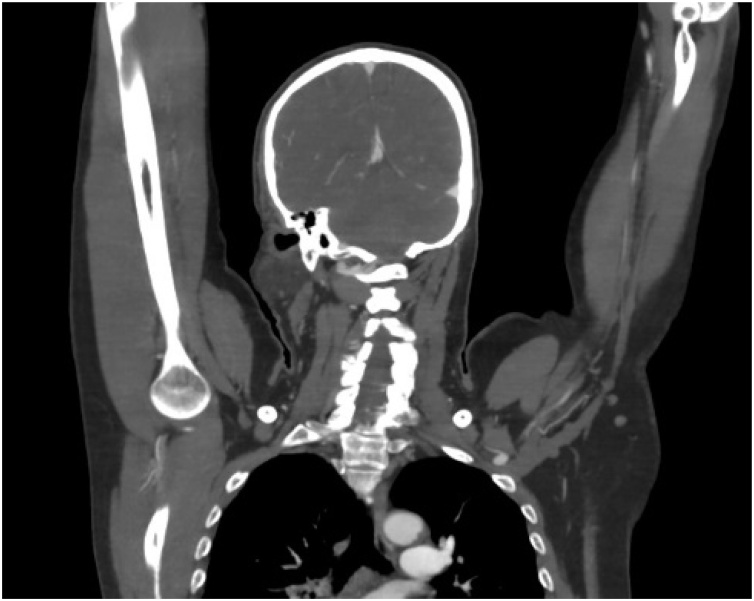
Left axillary node enlargement (white arrow) detected by chest CT scan including axilla with contrast at arterial phase.

A 65-year old man presented to our hospital eight years ago with a scar-like lesion at his left hand’s middle finger, specifically around proximal interphalangeal joint, he had noticed the symptoms for about 6 months prior to visit to the hospital. There was no previous history of burn or traumatic event. And also, no inflammation was noted. The lesion caused his affected joint to a limited range of motion and contracture deformity. As pathologic result from incisional biopsy confirmed MAC, the patient was then treated with ray amputation, a removal of the entire digit and greater part of its metacarpal bone. Pathologic report confirmed a complete tumor excision with free margin. Namely, there was no malignant cells seen within 2 mm or more from the tissue edge. Following a six-month interval surveillance, he had no symptoms until 8 years later when there was a presence of scar changes. The scar at the amputated stump had become harder and more contracted into the proximal part (Fig. 1A). At routine examination, a palpable round shaped nodule at medial side of his left elbow was found (Fig. 1B). An incisional biopsy was performed at the surgical scar and confirmed the diagnosis of MAC. Computed tomography (CT) scan of chest including axillary part was then performed and revealed lymph nodes enlarged not only at left elbow but also at left axillary region (Fig. 2). A provisional diagnosis of recurrent MAC with distant lymph node metastasis had been made. Consequently, left hand amputation and lymph node dissection at medial epicondyle and axillary region were performed. The pathologic examination confirmed that the malignant cells had metastasized to lymph nodes at both medial epicondyle and axillary regions. For this circumstance, as the diagnosis of a recurrence of the malignancy was made, the patient underwent an adjuvant radiation therapy. Currently, two years postoperatively, the patient remains in a complete remission.

## 3 Discussion

MAC was first introduced in 1982 as a rare locally aggressive malignancy of skin [1]. And it is increasingly rare among Asian population [8]. As its course is a slowly progressive disease, several morbidities usually reported are associated with local invasion of the disease and distant metastases are extremely rare [9]. With respect to this aspect, the disease’s course as metastasis to lymph nodes is unusual. In addition, generally the wide excision achieving tumor-free margin constitutes a definite treatment for cure [[3], [4], [5], [6]]. The presentation of our case that presented a recurrence with distant lymph node metastasis was unexpected.

The patient had a primary lesion arising at his hand, while MAC mostly affects adnexal structures particularly eccrine glands in the head and neck regions [2]. Therefore, everywhere where there are adnexal structures should be aware of the risk of this malignancy. Sometimes, however, it is difficult to differentiate MAC from other benign adnexal neoplasms [9]. Since this malignant tumor behaves insidiously and progresses gradually, this might explain why the patient survived uneventfully for 8 years following the surgery.

The patient also had had lymph node enlargement at his elbow during the examination. Consequently, although we could not palpate any nodules at the axillary region, he was then scheduled for chest CT scan including axilla to evaluate whether this part was actually negative for malignancy. Fortunately, we could detect the clinically negative nodes by this imaging technique. As a result, we suggested that CT scan plays an important role in such a suspicious situation. Wide local excision (WLE) is the most commonly performed treatment in MAC. But, due to an aggressive infiltrative nature of the disease, WLE was reportedly associated with high rate of positive surgical margin that require re-operation and local recurrence rate as high as 60% in some literature [10]. A plausible explanation for the recurrence in this patient might be that the complex structure of a hand, the malignant cells could also spread via tendon, sheath or palmar bursa that may require wider excision than that in the head and neck regions. In addition, Mohs micrographic surgery, a precise form of tissue sparing with microscopically controlled surgery, was reported to have much lower recurrent rate (0–12%) [11,12]. This may be due to the technique of surgery that requires examination of all margins of the tumor. Retrospectively, this surgical technique might help improve the outcome and prevent such recurrence. Unfortunately the technique was beyond our knowledge at that time.

As the circumstance was a recurrence of the malignancy, only amputation might be inadequate. Although the postoperative chemotherapy had shown less benefit [13], adjuvant radiation therapy is suggested in selected cases who had advanced MCANs [5]. Postoperatively, the patient underwent a course of radiation. To date, the patient is still alive and living uneventfully 2 years after surgery. A literature review found only a case of MCANs in which the malignancy had metastasized from scalp to gastrointestinal tract has been published in 2017 [14]. To our knowledge, the disease course of MAC with metastatic route through the lymphatic system like this case has never been reported before in the literature.

## 4 Conclusion

MAC arising at a hand, a structure more complex than the head and neck region, may require wider excision or some specific techniques in order to achieve the best result. Although the malignancy is an insidious and slowly progressive disease, distant metastasis should be kept in mind if there are some clinically suspicious conditions. Finally, CT scan will help render more details and make more precise diagnosis in a suspicious situation.

## Funding

This work received no funding.

## Ethical approval

The consent form and information sheet using in the process of obtaining a consent were approved by IRB at our institution.

## Consent

The patient has been informed prior to the conduction of this manuscript and informed consent has also been obtained. A copy of the written consent is available for review by the editor-in-chief of the journal on request.

## Author’s contribution

Kobkool Chakrapan Na Ayudhya and Vichack Chakrapan Na Ayudhya collected data and wrote manuscript.

Panat Tipsuwannakul, Sarun Thongvitokomarn, Vorapatu Tangsirapat, Panutchaya Kongon, Juthamas Thananon and Sirirat Sookpotarom contributed to conceptualization.

Paiboon Sookpotarom contributed to conceptualization, data curation, supervision and editing of the manuscript.

Paisarn Vejchapipat finally edited this manuscript.

## Registration of research studies

NA.

## Guarantor

Paiboon Sookpotarom.

## Provenance and peer review

Not commissioned, externally peer-reviewed.

## Declaration of Competing Interest

The authors declare that there is no conflict of interest regarding the publication of this article.
